# Continuous Non-Invasive Arterial Pressure Monitoring (ClearSight System) and Ankle Blood Pressure Measurements as Alternatives to Conventional Arm Blood Pressure

**DOI:** 10.3390/jcm9113615

**Published:** 2020-11-10

**Authors:** Seohee Lee, Jaeyeon Chung, Jinyoung Bae, Youn Joung Cho, Karam Nam, Yunseok Jeon

**Affiliations:** Department of Anesthesiology and Pain Medicine, Seoul National University Hospital, Seoul 03080, Korea; leesen34@gmail.com (S.L.); jychung1991@gmail.com (J.C.); baejy88@gmail.com (J.B.); mingming7@gmail.com (Y.J.C.); karamnam@gmail.com (K.N.)

**Keywords:** blood pressure, continuous non-invasive blood pressure, hemodynamic, monitoring

## Abstract

Measuring blood pressure (BP) via a pneumatic cuff placed around the arm has long been the standard method. However, in clinical situations where BP monitoring at the arm is difficult, the ankle is frequently used instead. We compared continuous non-invasive blood pressure (CNBP) measurements obtained at the finger, ankle BP and arm BP in patients undergoing breast cancer surgery. Arm BP, ankle BP (both obtained with a conventional pneumatic cuff) and CNBP measurements were obtained every 2.5 min during surgery. Correlation and Bland–Altman analyses were performed and differences among measurements were analyzed using a linear mixed model. A total of 245 sets of BP measurements were obtained from 10 patients. All systolic blood pressure (SBP), diastolic blood pressure (DBP) and mean blood pressure (MBP) measurements of ankle BP and CNBP were positively correlated with the arm BP measurements (Spearman rho 0.688–0.836, *p* < 0.001 for each correlation). The difference between CNBP and arm SBP was significantly smaller (least squares mean (95% confidence interval): −6.03 (−11.40, −0.67)) compared to that between ankle and arm SBP (least squares mean (95% CI): −15.32 (−20.69, −9.96), *p* = 0.019). However, this significant difference was not observed in DBP and MBP (−1.23 vs. 1.75, *p* = 0.190 and −3.85 vs. −2.63, *p* = 0.604, respectively). Ankle SBP measurements showed larger differences from arm SBP measurements than did CNBP SBP measurements in patients undergoing breast cancer surgery. CNBP could serve as a useful alternative to ankle BP when standard arm BP measurements cannot be obtained.

## 1. Introduction

Blood pressure (BP) is an essential hemodynamic parameter [1]. The gold standard method for BP monitoring is an oscillometric pneumatic cuff around the arm [2,3]. However, in some clinical situations, such as in cases with trauma of the arm, arteriovenous fistula or when the surgical site is on the arm, it can be difficult or impossible to monitor BP at the arm [3]. BP monitoring using a pneumatic cuff is generally avoided on the surgical-side arm of breast cancer patients due to concerns about lymphedema of the arm [4]. Moreover, intravenous catheters are usually placed on the non-surgical-side arm, where repeated BP measurements using a pneumatic cuff may cause occlusion of the intravenous line. Therefore, BP is frequently measured at the leg during breast cancer surgery [5].

However, leg BP readings can be higher than those of standard arm BP [3,6]. This results from pulse pressure amplification phenomenon where pulse pressure progressively increases from central arteries to peripheral arteries [7]. Therefore, hemodynamic management based on leg BP may result in hypotension. Intraoperative hypotension can lead to heart, kidney and nervous system complications [8,9]. Moreover, BP monitoring using an arterial catheter may be too invasive in many clinical situations. Therefore, there is a need for a non-invasive BP monitoring technique, as an alternative to leg BP and invasive arterial BP monitoring.

The ClearSight^TM^ system (Edwards Lifesciences Corp., Irvine, CA, USA) allows for continuous non-invasive blood pressure (CNBP) measurements to be obtained at the finger. The system uses the volume-clamp method where the changes in cuff pressures to maintain constant arterial transmural pressure are converted to brachial arterial pressure [10]. These procedures are performed throughout the whole cardiac cycle, allowing continuous blood pressure monitoring [10]. The ClearSight system has been evaluated in various clinical situations [11,12,13,14], and its use is known to be associated with a lower rate of hypotensive events [14,15,16].

The finger arteries are closer to the brachial arteries than the arteries of lower extremities and the ClearSight system has an intrinsic algorithm to reconstruct brachial pressure [10]. Additionally, the system does not interfere with peripheral intravenous catheter placement. Therefore, the ClearSight system can be an attractive alternative to leg BP as the system can be expected to show values closer to that of arm BP compared to leg BP. We hypothesized that, as a substitute for standard arm BP, CNBP would be a feasible alternative to ankle BP. To evaluate our hypothesis, we compared arm and leg BP measurements, obtained using a conventional pneumatic cuff, with CNBP measurements obtained using the ClearSight system in patients undergoing breast cancer surgery under general anesthesia.

## 2. Materials and Methods

### 2.1. Study Population

The study complied with the Declaration of Helsinki and the study protocol was approved by the Institutional Review Board of Seoul National University Hospital (No. 1906-113-1041). After obtaining informed consent, we enrolled patients with an American Society of Anesthesiologists physical status of 1–2 who were undergoing elective breast surgery under general anesthesia. The exclusion criteria were as follows: no palpable radial artery pulse, arteriovenous shunt, cardiac arrhythmia (including atrial fibrillation), peripheral vascular disease, trauma or skin disease in the arm or leg designated for BP measurement and requiring invasive arterial pressure monitoring.

### 2.2. Anesthesia

The patients were anesthetized according to our institutional protocol. Standard monitoring included electrocardiogram, pulse oximetry, temperature probe, bispectral index monitoring, end-tidal carbon dioxide monitoring and a non-invasive BP cuff was applied. Anesthesia was induced with 1.5–2 mg/kg propofol, 1–2 µg/kg fentanyl and 0.6–0.8 mg/kg rocuronium. The supraglottic airway was intubated and the lungs were ventilated with volume-controlled ventilation at a tidal volume of 6–8 mL/kg and respiration rate of 10–20/min, to titrate the end-tidal carbon dioxide pressure to 35–45 mmHg with a fraction of inspired oxygen of 0.5. Anesthetic agents were adjusted with 2.0–2.5 vol/% sevoflurane to maintain the bispectral index between 40 and 60. During surgery, the patients were positioned in a supine position with the surgical-side arm in an arm-positioning device.

### 2.3. Blood Pressure Monitoring

After measuring the circumference of the arm or ankle, an appropriately sized non-invasive BP cuff (Flexiport^®^; Welch Allyn Inc., Skaneateles Falls, NY, USA) was fitted. The bladder of the cuff was at least 40% of the circumference of the arm or ankle. The cuff was attached to the arm contralateral to the operation site, where a venous catheter was inserted. The middle of the bladder was placed over the brachial artery.

**Figure 1:**
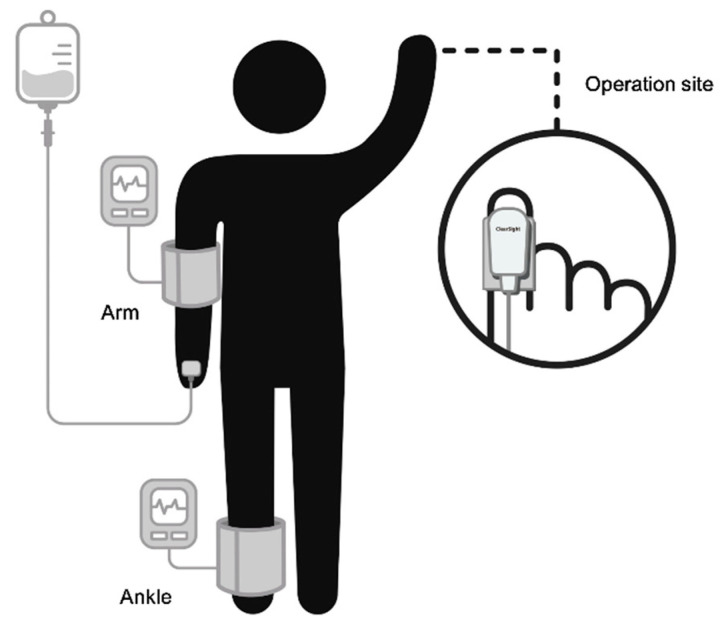
The ClearSight system is applied on a disposable cuff positioned over ipsilateral operation side arm and non-invasive blood pressure cuff is placed at the opposite arm and ankle.

Leg BP was measured at the ankle based on our experience and a previous study [3], in which measuring BP at the ankle was reportedly more comfortable than measuring it at the calf. Thus, a non-invasive BP cuff was placed on the ankle (Figure 1). The BP cuff was placed at the midpoint of the bladder over the posterior tibial artery, which is situated towards the lower end of the bladder (3 cm above the medial malleolus) [17]. The middle of the bladder was placed over the posterior tibial artery, which is located posterior to the medial malleolus.

The ClearSight system measures finger arterial pressure via an inflatable cuff fitted around the intermediate phalanx. The cuff was fitted to the index finger on the ipsilateral side of the operation site, contralateral side of the arm with the venous catheter. Measurements were obtained after zero calibration, performed according to the manufacturer’s protocol.

Systolic blood pressure (SBP), diastolic blood pressure (DBP) and mean blood pressure (MBP) were recorded every 2.5 min during surgery at the arm, ankle and finger.

### 2.4. Statistics

The sample size of 225 pairs of values was required to compare CNBP and arm BP with a power of 90% and an alpha level of 0.05, based on the expected mean bias of 2 mmHg and standard deviation (SD) of 9 mmHg reported by the previous review on the accuracy of the finger cuff [18] and the maximum allowed difference of 23 mmHg, which is about half the range of 95% limits of agreement between ankle and arm BP [3,19]. As usual breast cancer surgery in our center takes about 1 h, each patient was expected to have 24 pairs of values measured. Therefore, at least 10 patients were required to be enrolled.

To compare the relationship between each method, correlation coefficients were obtained considering the repeated measurement data [20]. We calculated bias, SD and limits of agreement (LOA) between the methods for each measurement of SBP, DBP and MBP, respectively, with the Bland–Altman method for repeated measures [21]. Since these data were measured several times from one person, the limits of agreement have been calculated to reflect this [22]. Bland–Altman analysis was carried out to demonstrate the bias (difference between arm BP and ankle BP, difference between arm BP and CNBP), precision (SD) and 95% limits of agreements (bias ± 2SD) between two measurement methods respectively. Mean values of differences of ankle BP from arm BP and CNBP from arm BP were compared using a linear mixed model. Statistical analyses were performed using Microsoft Excel 2012 (Microsoft Corporation, Redmond, Washington, DC, USA), SPSS version 25 (IBM SPSS statics, IBM Corporation, Somers, NY, USA), MedCalc Statistical Software version 19.1 (MedCalc Software bv, Ostend, Belgium) and SAS version 9.4 (SAS Institute, Cary, NC, USA).

## 3. Results

Ten patients were enrolled in, and completed, the study. Among the patients, nine received breast-conserving surgery (three right and six left), while the remaining patient received a total mastectomy. The demographic data of the enrolled patients are summarized in Table 1. A total of 245 sets of BP measurements were finally analyzed. Each BP measurement set comprised arm BP, ankle BP and CNBP measurements (Table 2).

**Figure 2:**
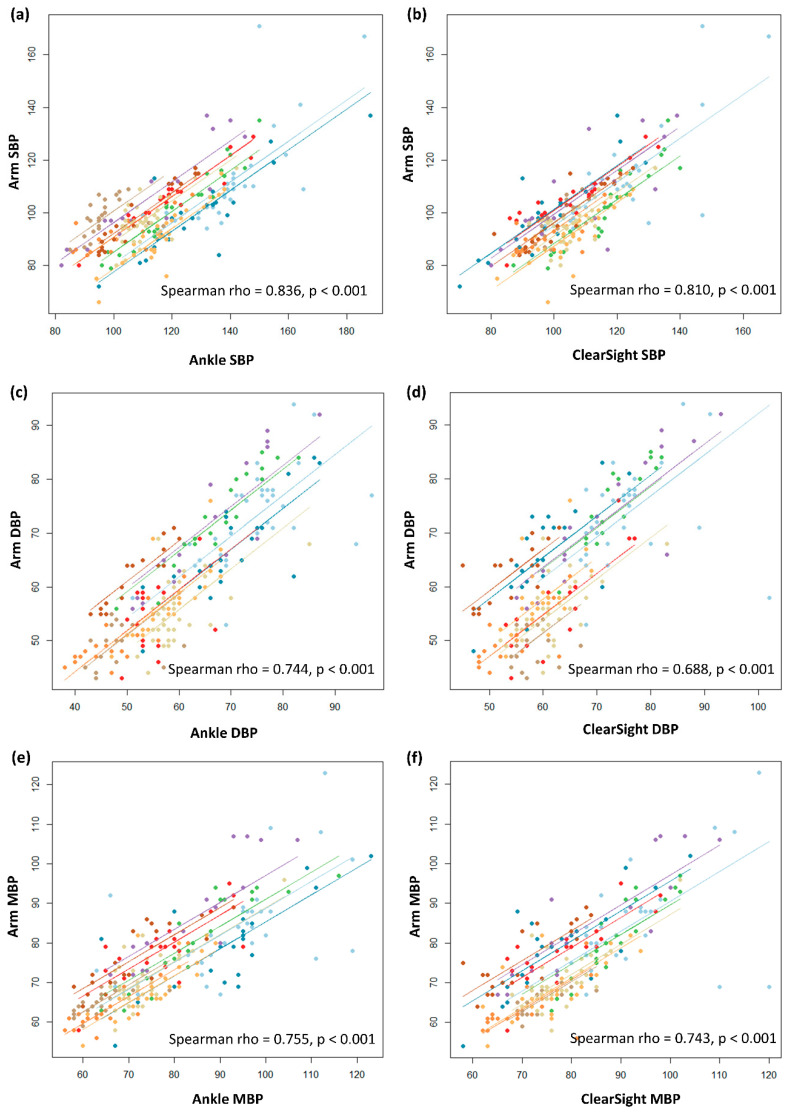
The scatterplots for correlation of blood pressure measurements. (**a**) Correlation between systolic blood pressure (SBP) of arm and ankle. (**b**) Correlation between SBP of arm and ClearSight. (**c**) Correlation between diastolic blood pressure (DBP) of arm and ankle. (**d**) Correlation between DBP of arm and ClearSight. (**e**) Correlation between mean blood pressure (MBP) of arm and ankle. (**f**) Correlation between MBP of arm and ClearSight. Each color of the dots and the regression lines represents each patient.

The ankle SBP, DBP and MBP measurements were positively correlated with those from the arm (Spearman rho = 0.836, *p* < 0.001; 0.744, *p* < 0.001; and 0.755, *p* < 0.001, respectively). The CNBP SBP, DBP and MBP measurements were positively correlated with the arm BP measurements (Spearman rho = 0.810, *p* < 0.001; 0.688, *p* < 0.001; and 0.743, *p* < 0.001, respectively; Figure 2).

**Figure 3:**
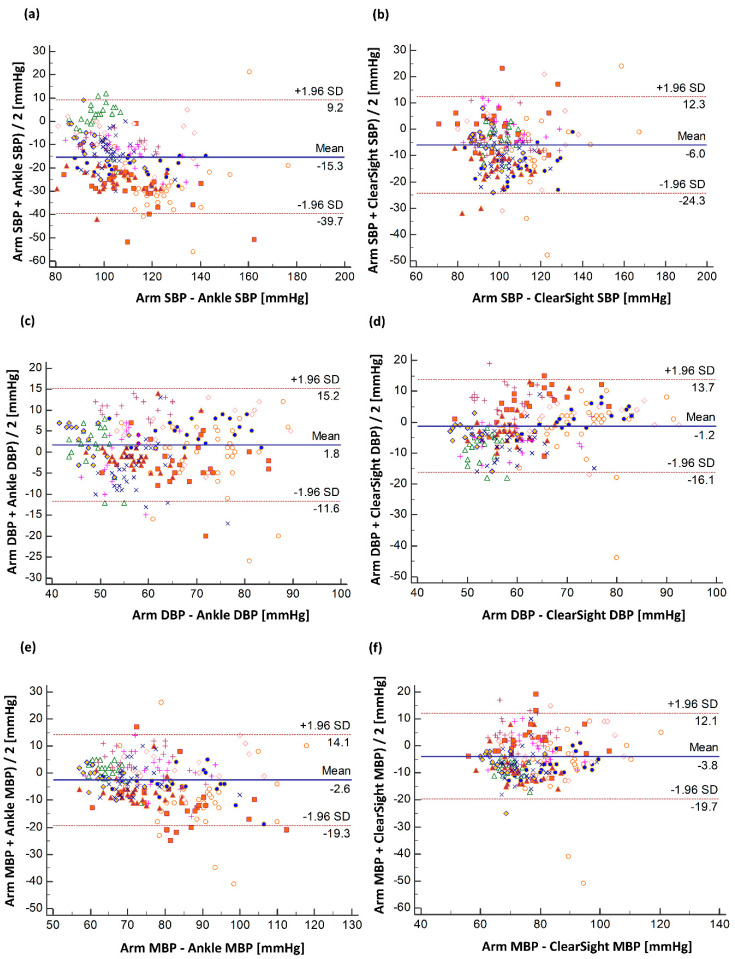
Bland–Altman plot for BP measurements. (**a**) Bland–Altman analysis between systolic blood pressure (SBP) of arm and ankle. (**b**) Bland–Altman analysis between SBP of arm and ClearSight. (**c**) Bland–Altman analysis between diastolic blood pressure (DBP) of arm and ankle. (**d**) Bland–Altman analysis between DBP of arm and ClearSight. (**e**) Bland–Altman analysis between mean blood pressure (MBP) of arm and ankle. (**f**) Bland–Altman analysis between MBP of arm and ClearSight. Each color of dot represents the measurements from each patient.

Bland–Altman analysis showed that the mean ± SD difference (95% limits of agreement) between arm and ankle BP measurements was −15.3 ± 12.4 mmHg (39.7 to 9.2) for SBP, 1.8 ± 6.8 mmHg (−11.6 to 15.2) for DBP and −2.6 ± 8.5 mmHg (−19.3 to 14.1) for MBP (Figure 3a,c,e); the respective differences between arm BP and CNBP measurements were −6.0 ± 9.3 mmHg (−24.3 to 12.3), −1.2 ± 7.6 mmHg (−16.1 to 13.7) and −3.8 mmHg ± 8.1 mmHg (−19.7 to 12.1 mmHg) (Figure 3b,d,f).

**Figure 4:**
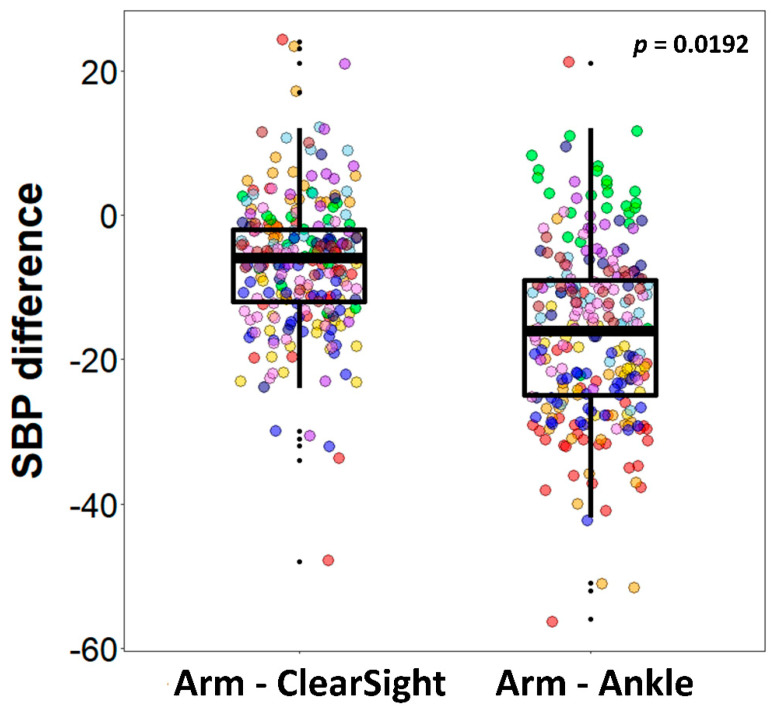
The boxplot showing the differences of CNBP SBP and ankle SBP from arm SBP. Each color of dots represents each patient.

**Figure 5:**
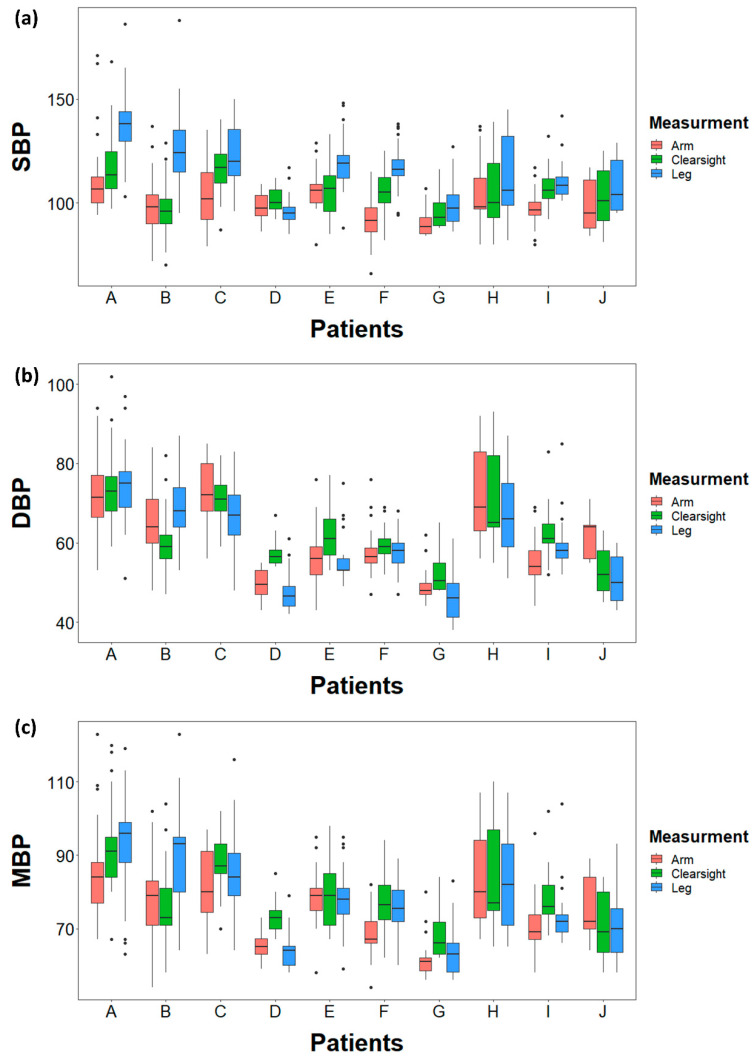
The boxplots of each individual patient showing distribution of (**a**) SBP, (**b**) DBP and (**c**) MBP measurements by each of three methods. The red boxes represent measurements from arm, the green boxes represent measurements from ClearSight and the blue boxes represent measurements from ankle.

The difference between CNBP and arm SBP was significantly smaller (least squares mean (95% confidence interval): −6.03 (−11.40 to −0.67)) compared to that between ankle and arm SBP (LS mean (95% CI): −15.32 (−20.69 to −9.96), *p* = 0.019) (Table 3 and Figure 4). However, this significant difference was not observed in DBP and MBP (−1.23 vs. 1.75, *p* = 0.190 and −3.85 vs. −2.63, *p* = 0.604, respectively). The differences between the arm BP, ankle BP and CNBP values for each of the 10 subjects are presented in Figure 5.

No adverse events were observed during the study, including any signs of pressure-induced damage from the finger cuff.

## 4. Discussion

In this study, CNBP (ClearSight system) and ankle BP measurements were both correlated with arm BP measurements in patients undergoing breast cancer surgery. However, the difference between ankle and arm SBP was significantly greater than that between CNBP and arm SBP. No such significant differences were observed for MBP or DBP.

Arm BP obtained with a pneumatic cuff has been the gold standard BP measurement method in clinical practice [23]. Most guidelines and recommendations are based on this BP measurement method [23,24]. However, obtaining BP measurements at the arm with a pneumatic cuff is not always feasible, including during breast cancer surgery. In patients undergoing such surgery, BP monitoring using a pneumatic cuff on the surgical-side arm is avoided to prevent lymphedema [4]. Many studies have evaluated the feasibility of obtaining BP measurements at other sites as a substitute for standard arm BP [3,15,25,26,27].

BP measurements obtained at the lower extremities, including ankle BP, have been well studied [3,5,6,25,26,28,29]. Wilkes et al. [6] reported that the ankle is a suitable alternative site for BP measurement only when the arm is not available, because ankle BP measurements tend to be higher than brachial ones [3,6,25,26]. Proposed reasons for the higher leg BP readings include greater resistance of distal vessels, ineffective compression by the cuff on the posterior tibial artery and poor detection of the oscillations [26]. In addition, ankle BP measurements may be affected by positions, such as the lithotomy and reverse Trendelenburg positions [28,29].

A false high SBP may have clinical implications. During surgery under general anesthesia, patient management based on ankle BP could lead to hypotensive events due to unnecessary attempts to lower SBP using anti-hypertensive drugs or increasing the dosage of anesthetic agents. Although MBP has been regarded as a better indicator of perfusion of vital organs [30], a high SBP also influences the decisions of clinicians, such that high-dose anesthetic agents, opioids or antihypertensive drugs may be administered to manage a high SBP during general anesthesia.

Many studies have reported devices that can be used to obtain BP measurements as an alternative to conventional pneumatic cuff BP measurements, including the ClearSight system [11,12,13,15,16]. Good agreement between ClearSight MBP and DBP measurements and invasive arterial pressure measurements was observed in obese patients undergoing laparoscopic gastrectomy under general anesthesia, although for SBP the agreement was only moderate [13]. In addition, a randomized controlled study on caesarean delivery reported fair agreement between ClearSight and non-invasive BP measurements obtained using a conventional BP cuff under spinal anesthesia [14]. In other studies, CNBP measurements were associated with a lower incidence of hypotension during surgery compared to conventional pneumatic cuff measurements obtained at the arm [14,15,16]. However, no study has compared ClearSight and lower extremity BP values.

Several limitations of this study should be discussed. Firstly, the study was only performed on women undergoing breast surgery with a limited number of patients. Therefore, the results might not apply to other clinical settings, such as male patients or very old and dehydrated patients. Secondly, we enrolled only American Society of Anesthesiologists physical status 1–2 patients who were undergoing breast cancer surgery under general anesthesia. Additionally, we excluded patients with peripheral vascular diseases, such as peripheral atherosclerosis and autoimmune diseases affecting peripheral vessels. Thus, our results may not be applicable to patients under local anesthesia, awake patients or patients with comorbidities. Therefore, further studies may be needed to validate our results in other clinical populations. Thirdly, CNBP readings obtained using the ClearSight system could be a useful alternative to ankle SBP. In addition, the ClearSight system is less invasive compared to arterial catheter BP monitoring. Therefore, CNBP seems to be an attractive and feasible alternative to arm BP. However, compared to conventional pneumatic cuff BP monitoring systems, the ClearSight system is costly. Further studies should conduct cost-benefit analyses of the ClearSight system.

## 5. Conclusions

In conclusion, ankle BP and CNBP measurements were correlated with standard arm BP measurements in patients undergoing breast cancer surgery. Furthermore, ankle SBP measurements showed larger differences from standard arm BP measurements than did CNBP measurements. CNBP could serve as a useful alternative to ankle BP when standard arm BP measurements cannot be obtained.

## Figures and Tables

**Table 1:** Demographic features of patients.

Patients No	Age	Gender	Wight (kg)	Height (cm)	BMI (Kg/m^2^)	Operation Type
1	50	Female	55.45	156.9	22.52	Total mastectomy
2	76	Female	48.3	153.1	24.87	BCS,Lt sentinel bx
3	43	Female	57.65	162	21.97	BCS,Rt sentinel bx
4	57	Female	54.1	156.3	22.15	BCS,Lt sentinel bx
5	63	Female	58.85	155.4	24.37	BCS,Rt sentinel bx
6	62	Female	61	158.6	24.25	BCS,Lt sentinel bx
7	55	Female	60.9	154.5	25.51	BCS,Rt sentinel bx
8	48	Female	55.6	161.5	21.32	BCS,Lt sentinel bx
9	43	Female	57.3	164	21.3	BCS,Lt sentinel bx
10	71	Female	56.4	158.5	22.45	BCS,Lt sentinel bx

The 10 subjects had breast conserving surgery or total mastectomy. As reference to brachial noninvasive arterial pressure, both ankle non-invasive arterial pressure and ClearSight arterial pressure were measured and recorded. BCS, breast conserving surgery; BMI, body mass index; bx, biopsy; Lt, left; Rt, right.

**Table 2:** Mean and standard deviation values of systolic, mean and diastolic blood pressure (mmHg) measured by the three different methods.

Blood Pressure	Arm	Ankle	ClearSight
Systolic blood pressure (mmHg)	100.11 (13.93)	116.63 (18.45)	106.71 (14.04)
Diastolic blood pressure (mmHg)	61.31 (11.17)	60.30 (11.39)	62.92 (9.61)
Mean blood pressure (mmHg)	75.09 (11.61)	78.43 (13.98)	79.55 (11.41)

Data are presented as mean (SD).

**Table 3:** Difference between Arm–Ankle and Arm–ClearSight compared using a linear mixed model.

BP	Sites Compared	Least Squares Means (95% Confidence Interval)	Differences of Least Squares Means (95% Confidence Interval)	*p*-Value
SBP	Arm-Ankle	−15.32(−20.69,−9.96)	−9.29(−16.876,−1.704)	0.0192
	Arm-ClearSight	−6.03(−11.40,−0.67)		
DBP	Arm -Ankle	1.75(−1.15,5.00)	2.98(−1.61,7.57)	0.1899
	Arm-ClearSight	−1.23(−4.48,2.02)		
MBP	Arm-Ankle	−2.63(−6.06,0.79)	1.22(−3.63,6.06)	0.6043
	Arm-ClearSight	−3.85(−7.27,−0.43)		

BP, blood pressure; SBP, systolic blood pressure; DBP, diastolic blood pressure; MBP, mean blood pressure.
